# Sap flow of black locust in response to short-term drought in southern Loess Plateau of China

**DOI:** 10.1038/s41598-018-24669-5

**Published:** 2018-04-18

**Authors:** Qingyin Zhang, Xiaoxu Jia, Mingan Shao, Chencheng Zhang, Xiangdong Li, Changkun Ma

**Affiliations:** 1 State Key Laboratory of Soil Erosion and Dryland Farming on the Loess Plateau, Northwest A&F University, Yangling, 712100 China; 2 Key Laboratory of Ecosystem Network Observation and Modeling, Institute of Geographic Sciences and Natural Resources Research, Chinese Academy of Sciences, Beijing, 100101 China; 3 College of Resources and Environment, University of Chinese Academy of Sciences, Beijing, 100190 China

## Abstract

Soil water shortage is a major factor influencing the ecology and hydrology of vegetation in China’s semihumid Loess Plateau. However, few studies have experimentally assessed how expected changes in precipitation will affect sap flow in semihumid forest ecosystems. In this study, we measured the sap flow of black locust (*Robinia pseudoacacia* Linn.) under ambient and drought (induced by throughfall exclusion) conditions in 2015 and 2016, and investigated the relationship between stand transpiration and environmental factors in the semihumid China’s Loess Plateau. Throughfall exclusion significantly decreased sap flux density and stand transpiration by 39% and 28%, respectively, in 2016, which may have been due to the cumulative droughts effect from both 2015 and 2016. Throughfall exclusion caused a significant reduction in soil moisture, leaf area index (LAI), and stem diameter. Stand transpiration was positively correlated with LAI (*P* < 0.01), but precipitation and soil moisture did not correlate with stand transpiration at a daily timescale, suggesting that LAI can be used as a proxy for stand transpiration. Our results highlight that precipitation must be considered when planting black locust in semihumid regions. These findings provide basic information about the management of water resources and vegetation restoration in the semihumid China’s Loess Plateau and possibly other water-limited regions around the world.

## Introduction

As an area with high atmospheric evaporative demand and low rainfall^1^, the semihumid Loess Plateau is facing increasing water shortages^2^. Over the last 30 years, the climate has dramatically changed in the dominate drylands of north China, where soil moisture is a significant terrestrial water resource^3^. A series of large afforestation campaigns, including the ‘Grain for Green’ program (GFGP), were initiated by the Chinese government at the end of the 1990s^4^. These campaigns resulted in increasing carbon sequestration^5^, a reduction in water loss, and improved control of soil erosion^6^. Recent studies showed that large-scale afforestation operations such as the planting of black locust (*Robinia pseudoacacia* Linn.) can strongly affect the dynamics of soil moisture by changing the evapotranspiration, infiltration and surface runoff in theregion^7^. In the past, unsuitable reforestation has resulted in soil water deficits owing to the high water consumption of the planted trees and, consequently, has led to early degradation of the tree plantations^8^. Therefore, to encourage appropriate reforestation practices, the relationship between the tree growth and soil moisture needs to be investigated to determine if a given area is suitable for developing stable forest ecosystem using reforestation.

Drought tolerance is frequently addressed when evaluating appropriate reforestation practices in semi-arid areas^1^. To maintain the integrity of their carbon assimilation and hydraulic systems, plants will undergo structural or physiological adjustment when they are confronted with soil moisture stress^9^. The effects of drought stress on the physiology of black locust, such as changes to the developed root systems, photosynthesis, and stomatal control of transpiration, have been documented on the Loess Plateau^1,10–12^. However, there is limited information about the transpiration and sap flow characteristics of individual large trees in relation to soil moisture stress within a forest stand.

In recent years, plant water use has been measured using thermal dissipation probe (TDP) methods^1,13,14^. In areas subjected to drought, transpiration or sap flux measurements are particularly suitable for investigations into the different strategies that vegetation use to cope with limited soil water availability^15^. Kume *et al*.^16^ reported that sap flux density decreased significantly during the seasonal soil drought conditions for an evergreen tropical stand. A positive relationship between stand transpiration and soil moisture conditions was detected at an annual scale in the semi-arid region of the Loess Plateau^17^. Obviously, in areas with insufficient water, soil moisture conditions can restrict many physiological processes. For example, stem growth is often identified as the most sensitive indicator of the degree of drought stress because it is low on the carbon allocation hierarchy^18^. Wang *et al*.^19^ suggested that lower leaf area index (LAI) and total sapwood area would result in the relatively low transpiration in a growing season. However, we have a poor understanding of the responses of these variables to drought in large trees in the semihumid Loess Plateau in China.

Black locust is an exotic fast-growing species very common to the regions southern of semihumid Loess Plateau. However, soil desiccation has caused degradation of black locust forest plantations whereby mature trees have developed abnormally short trunks^20,21^. Therefore, with wide implementation of GFGP, it was hypothesized that the sap flow and growth of black locust would be inhibited with the intensification of soil moisture stress despite of its characteristic of drought tolerance. The objectives of this study were: (1) to estimate the whole-tree transpiration, stem growth, and LAI of black locust under different soil moisture conditions (“drought” soil moisture conditions, implemented with throughfall exclusion, and “ambient” soil moisture conditions), and (2) to compare the relationships among biological, edaphic, and environmental characteristics.

## Results

### Soil moisture

**Figure 1:**
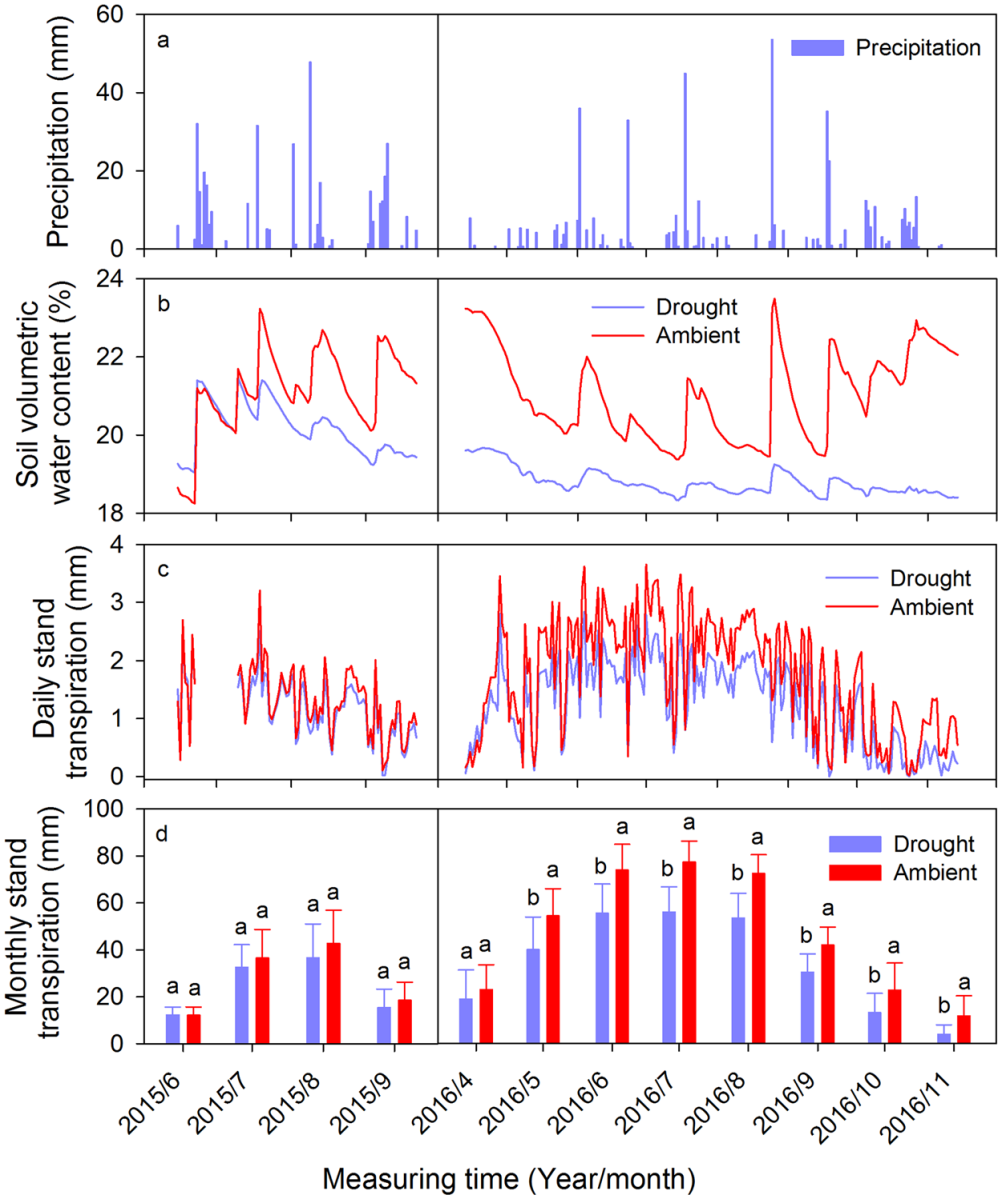
Temporal variation in measured values of precipitation (**a**), soil volumetric water content (0–1 m) (**b**), daily stand transpiration (**c**), and monthly stand transpiration (**d**) at the study site between June 2015 and November 2016 in the drought and ambient plots. Significant differences between drought and ambient plots within the same time are denoted by different lowercase letters. Values of monthly stand transpiration represent the mean ± SD for a sample size of n = 6. In June 2015, sap flow density was measured from June 10 to June 25, so the monthly stand transpiration in June was estimated proportionately. The total precipitation is 815 and 556 mm in 2015 and 2016, respectively. In the drought treatment, the throughfall is 326 and 222 mm in 2015 and 2016, respectively.

Soil moisture at depths of 0–1 m was significantly decreased in 2016 due to throughfall exclusion (*P* < 0.05, Fig. 1b). The daily average soil moisture in the ambient treatment fluctuated corresponding to the occurrence of rainfall events, whereas it decreased gradually over time under the throughfall exclusion treatment. The soil volumetric water content was 20.1 and 18.7% in the throughfall exclusion treatment in 2015 and 2016, respectively at 0–1 m soil depths, while soil moisture was 20.9 and 21.4% in the ambient treatment 2015 and 2016, respectively.

### Leaf area index and stem growth

**Figure 2:**
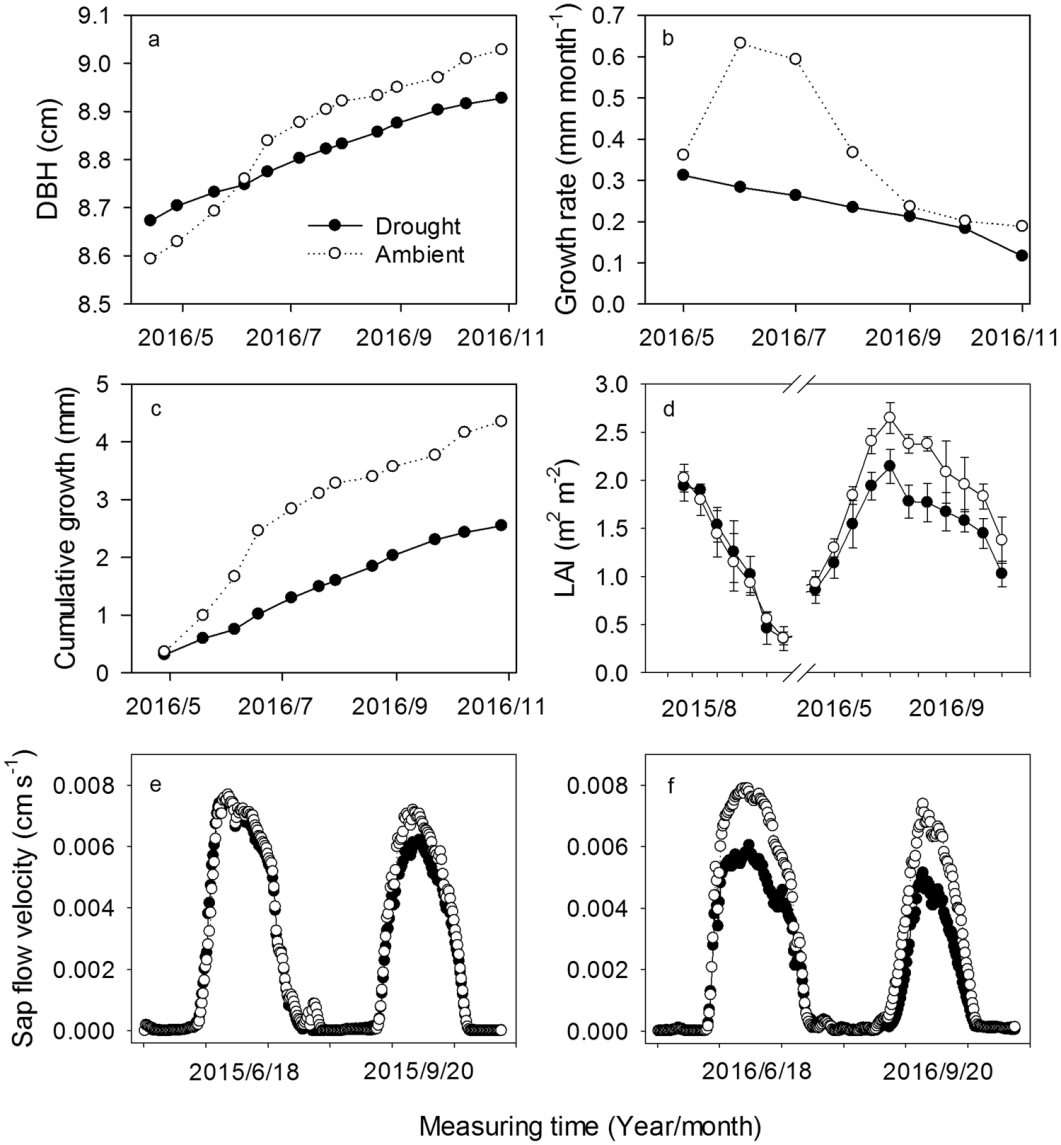
Temporal variation in measured values of stem growth (**a**,**b**, and **c**), leaf area index (LAI) (**d**), and diurnal sap flow velocity (e and f) in the drought and ambient plots at the study site.

Leaf area index (LAI) under the two treatments was obtained for each month during the growing seasons, and ranged from 0.36 to 2.65 (Fig. 2d). Throughfall exclusion caused a significant reduction in LAI compared with the control in 2016 (*P* < 0.05). In 2015, there was no difference in LAI between treatments, however, LAI decreased by 21% after throughfall exclusion compared to the ambient treatment in 2016.

Throughfall exclusion reduced stem diameter growth of individuals compared with the control in 2016 (Fig. 2a). Stem diameter grew less in the throughfall exclusion treatment (2.5 mm yr^−1^) than in the ambient treatment (4.3 mm yr^−1^) in 2016. From June to August, stem growth in the ambient treatment (0.53 mm month^−1^) was higher than stem growth in the throughfall exclusion treatment (0.26 mm month^−1^) (Fig. 2b).

### Sap flux density and stand transpiration

Figure 2c,d shows the diurnal courses of average sap flux density under the two treatments on four typical bright days during the growing season. Sap flux density was approximately zero between 0:00 and 6:00 h, and increased shortly after sunrise, reached a maximum density between 12:00 and 14:00 hours, and then decreased in the late afternoon. Throughfall exclusion significantly decreased sap flux density compared with the control in 2016 (*P* < 0.05).

**Figure 3:**
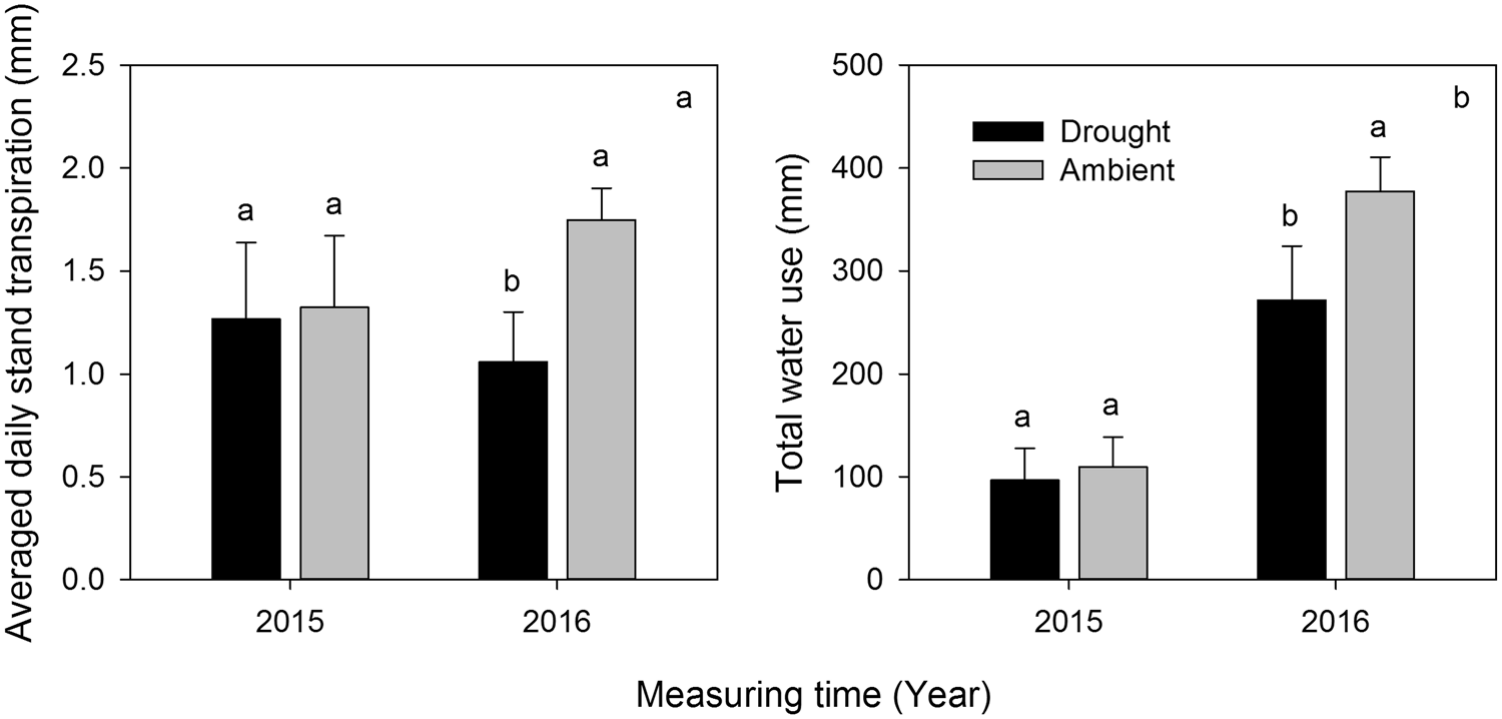
Averaged daily stand transpiration (**a**) and total water use (**b**) measured for black locust stands in 2015 and 2016 in the drought and ambient plots at the study site. Note: in 2015, we only measured the sap flow from June 14 to September 22 due to the technical problems. Therefore, the total water use in 2015 was calculated by using the sap flow data from June 14 to September 22. The total water use in 2016 was calculated by using the sap flow data from April 14 to November 14.

Daily stand transpiration (Qd) was obtained for each measurement day. The Qd ranged from 0 to 2.84 mm, and exhibited pronounced diurnal variations (Fig. 1c). In 2015, the average Qd (1.27 mm day^−1^) in the throughfall exclusion treatment was similar to that in the ambient treatment (1.32 mm day^−1^). In 2016, the average Qd (1.06 mm day^−1^) in the throughfall exclusion treatment was significantly lower than in the ambient treatment (1.75 mm day^−1^) (*P* < 0.01) (Fig. 3a). The monthly stand transpiration (Qm) in the throughfall exclusion treatment (34 mm month^−1^) was significantly lower than in the ambient treatment (47 mm month^−1^) (*P* < 0.01, Fig. 1d). The maximum Qm values occurred in July during both 2015 and 2016, and decreased gradually throughout the late growing season. During the 2015 measurement period, the total stand transpiration in the throughfall exclusion treatment and the ambient treatment were 97 and 110 mm, respectively. In 2016, the total stand transpiration in the throughfall exclusion treatment and the ambient treatment were 272 and 378 mm, respectively (Fig. 3b).

### Stand transpiration response to environmental variables

**Figure 4:**
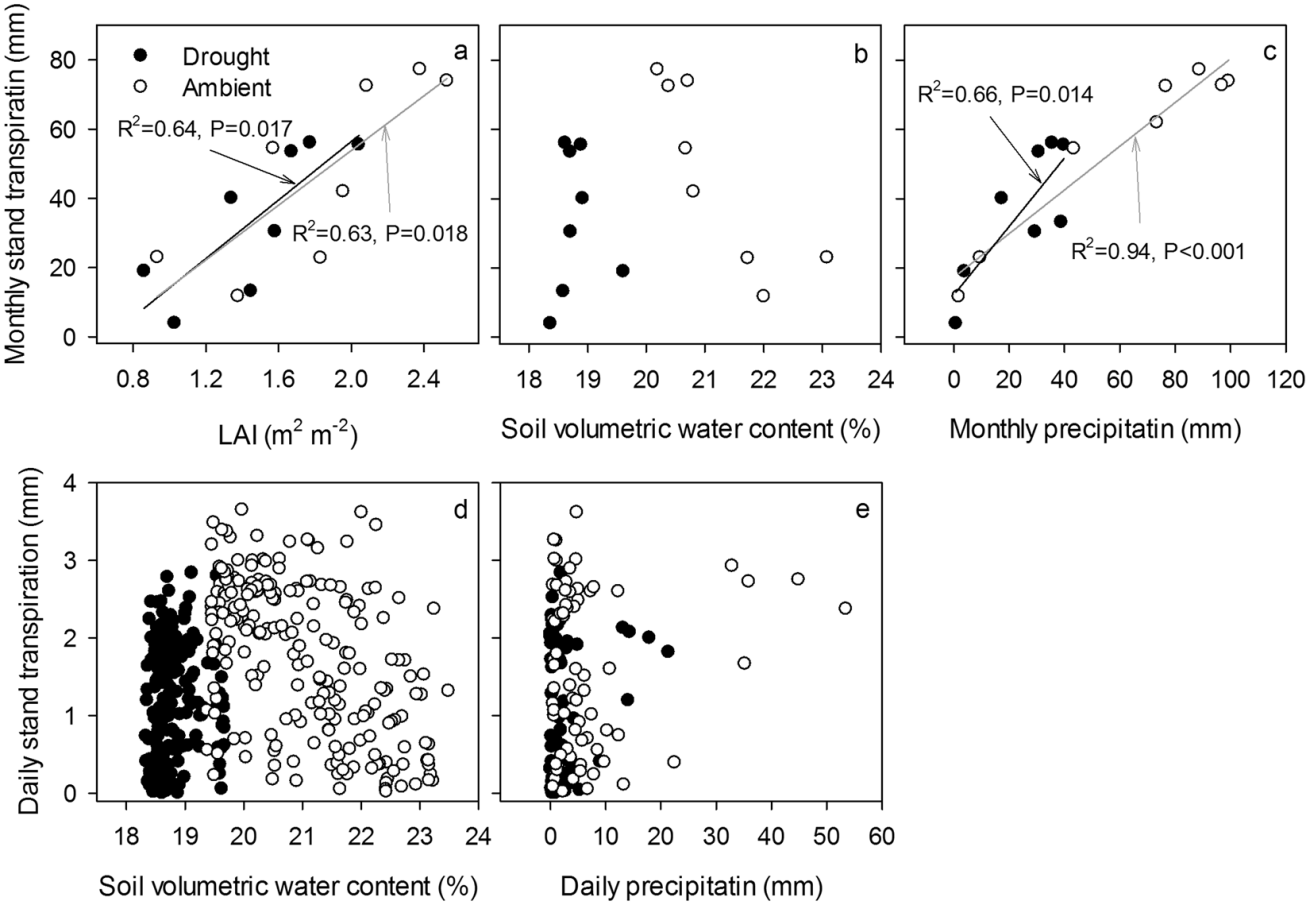
Relationships between stand transpiration and leaf area index (LAI) (**a**), soil volumetric water content (**b**,**d**), and precipitation (**c**,**e**). The figure was drawn based on the data of 2016.

The relationships among stand transpiration, LAI, soil moisture, and precipitation are presented in Fig. 4. Linear regression of Qm with LAI were established for throughfall exclusion (Qm = 0.015 * LAI + 0.953, R² = 0.64, *P* < 0.01) and ambient (Qm = 0.016 * LAI + 1.075, R² = 0.63, *P* < 0.01) treatments (Fig. 4a). Linear regression of Qm with monthly precipitation were established for throughfall exclusion (Qm = 0.462 * P + 8.813, R² = 0.38, *P* < 0.05) and ambient (Qm = 0.949 * P + 16.485, R² = 0.41, *P* < 0.05) treatments (Fig. 4c). There was a significant negative correlation between monthly stand transpiration and monthly soil VWC at 0–1 m depths (Fig. 4b). However, there was no significant relationship among stand transpiration, daily soil VWC, and daily precipitation (Fig. 4d,e). In addition, stepwise regression analysis showed that precipitation was the main factor in the ambient treatment and when data from both treatments was considered (all data) while LAI was the main factor in the drought treatment (Table 1).

**Table 1:** Stepwise regression results to detect factors (monthly precipitation, P; and leaf area index, LAI) determining monthly stand transpiration in the drought and ambient plots.

Treatment	Equation	R^2^	Sig. (P)	n
Ambient	Qm = −22.4 P + 522.2	0.717	<0.001	8
Drought	Qm = 42.3LAI − 28.1	0.642	<0.001	8
All	Qm = 39.9LAI −25.2	0.666	<0.001	16

## Discussion

### Estimation of stand transpiration

Scaling up the water use from individual trees to stand level is a straightforward approach^14,17,19^. In our study, the daily mean transpiration of black locust grown under ambient conditions was 1.75 mm day^−1^ and total water use in the ambient condition was 378 mm in 2016. Only few studies have been conducted on seasonal water use of black locust trees in China’s Loess Plateau region, the results of which are in agreement with our results. For example, Wang *et al*.^19^ noted total growing seasonal water use rates of 0.35 mm day^−1^ for 30-year-old black locust stands. Zhang *et al*.^17^ observed water use rates of 0.44 mm day^−1^ in the growing season in 2008, and 0.30 mm day^−1^ in 2009, for 30-year-old stands. Jiao *et al*.^22,23^ found that water use rates of 12-year-old and 28-year-old stands ranged from 0.14 mm day^−1^ to 0.23 mm day^−1^ in the growing season in 2014. The stand transpiration was consistent with those recorded by other studies of broadleaf trees^14,24,25^. It has been reported that water use through transpiration in temperate deciduous forests is 300–350 mm during a complete growing season^26,27^, which was consistent with the plantation in this study. Black locust in this stand transpired 63% of potential evapotranspiration during the full-leaf periods, and the ratio was higher than those of previous reports of over 30%^13,28^.

The large discrepancy between our result and those of the other studies was likely due to differences in the average sap flux density of the stand and the stand sapwood area^13,14,17,19^. First, since the thickness of black locust sapwood was relatively narrow, ranging from 7 to 12 mm, we considered a 10-mm probe acceptable for estimation of whole-tree sap flow, and radial variation in sap flux density was neglected in this study. Second, as part of the calibration process, a miscalculation of zero sap flow may lead to the under- or over-estimation of transpiration^13^. In the present study, zero flow was assumed to occur at nighttime (02:00 and 05:00) when sap flow is assumed to be negligible. Third, technical problems may be another source of error in transpiration estimates. Transpiration estimated using TDP method appeared lower than from eddy covariance or catchment water balance^29^. Therefore, to obtain accurate estimates of transpiration, species-specific and model coefficient calibration (e.g., on cut tree experiments) could increase its reliability in this region. Furthermore, other possible reasons for the discrepancy may include physiological (e.g., species and vessel density) and topographic aspects (e.g., slope direction)^30^.

### Stand transpiration in relation to variables

To further elucidate the response patterns of stand transpiration to environmental factors for black locust, data sets of daily or monthly transpiration vs. LAI, soil moisture, and precipitation were analyzed from the ambient and drought conditions, respectively (Fig. 4). As the main factors that affect plant growth in semi-arid regions, soil moisture and related factors (precipitation) generally affect water consumption by transpiration and water potential^31^. Several researchers have reported that soil moisture strongly affects the variation of transpiration. For example, Chang *et al*.^32^ established a logistic functional relationship between daily transpiration and soil water content, which explained 84% of the variation in transpiration. Normalized sap flux density of black locust showed a high sensitivity to soil moisture conditions and monthly precipitation^1^. Black locusts are considered drought sensitive due to their drastic reductions or increases in transpiration in response to soil moisture changes^1^. However, in our study, no correlations were detected between daily stand transpiration and daily soil VWC or daily precipitation (Fig. 4). These results are consistent with the findings that precipitation and soil VWC did not show a clear correlation with stand transpiration at a daily timescale^17,33^. This can be explained by the negative relationships between precipitation, and vapor pressure deficit, solar radiation and potential evapotranspiration^17^, that is, more rainy days result in less transpiration driving power. In addition, a higher mean annual precipitation in our study area (592 mm) than the other site (498 mm)^1^ was therefore most likely responsible for the difference in the relationship between soil moisture and stand transpiration between sites. However, at a monthly scale, we found positive correlations between monthly stand transpiration and precipitation (Fig. 4c), implying the influence of precipitation on transpiration at longer timescales. It has been reported that soil moisture conditions are critical to stand transpiration variations in tropical deciduous forests at longer timescale and can be a key factor in leaf flushing and abscission^33^. In our study, drought stress significantly decreased sap flux density and stand transpiration by 39% and 28% in 2016, respectively. However, the water was seriously limited in 2015 and throughfall exclusion further aggravated the drought in 2016. Therefore, a large proportion of reduced water use may be caused by the drought conditions during 2015 and 2016.

In 2016, total water use in the ambient treatment (378 mm) was significantly higher than in the throughfall exclusion treatment (272 mm). The differences in stand transpiration between the ambient and drought treatments may be related to the differences in tree morphology^19^. When black locust is confronted with drought stress, it will incur structural or physiological changes in order to maintain the integrity of the hydraulic system and to enable carbon assimilation despite substantial water losses^9^. For example, throughfall exclusion significantly reduced the LAI (Fig. 2d), which was positively correlated with stand transpiration (Fig. 4a). This is consistent with the opinion that leaf is the most important organ of transpired water^34^. In present study, the decreasing LAI in drought plots was observed for black locust in late 2016, probably as a result of soil drought. This might be one of the passive strategies to reduce water consumption for this species. Hara *et al*.^35^ also reported that black locust tends to drop leaves in response to soil drought. In addition, the slow growth of stem diameter under drought condition (Fig. 2a) may lead to the lower sapwood area under drought conditions compared to ambient conditions, resulting in lower stand transpiration. Furthermore, as vascular plants, black locust employ the cohesive forces between water molecules as a means of connecting transpiring leaves, via the xylem vascular system, to a reliable source of water in the soil^36,37^. When the plants are confronted with drought stress, hydraulic failure could crush cells and kill tissues in the xylem vascular system^38^, which play a key role in hydraulic conductance from soil to atmosphere. Therefore, the physiology of cambial cells and earlywood vessel in the xylem under drought stress should be further researched.

## Conclusions

This study investigated the discrepancy in transpiration for black locust stands in drought and ambient conditions on the Loess Plateau of China. Throughfall exclusion significantly decreased sap flux density and stand transpiration of black locust by 39% and 28%, respectively, in 2016. Throughfall exclusion significantly reduced soil VWC, LAI, and stem diameter. Monthly stand transpiration was positively correlated with LAI, indicating that LAI can be used as a proxy for stand phenology. Precipitation also showed a positive correlation with stand transpiration on a monthly timescale. However, precipitation and soil moisture did not show a clear correlation with stand transpiration at a daily timescale. The results from this study suggest that although the stand transpiration is controlled by multiple factors, the significant factors differ at different timescales. Despite having observed only two years of experiment, these findings provide basic information regarding a water use strategy for black locust when drought conditions are present in this semihumid area. Further research should be conducted to observe the multi-year transpiration on the Loess Plateau.

## Materials and Methods

### Study site

The study site, shown in Fig. S1, is located at Yehe National Forestry Center in the Qishui watershed (34.55°N, 107.90°E; 1080 m. a.s.l.), in Fufeng County, Shaanxi Province, situated on the southern Loess Plateau in China. The area is characterized as a semihumid, temperate continental climate zone. The mean annual precipitation is 592 mm, with 70% occurring from June to September. The mean annual temperature is 11.5 °C, ranging from −2 °C in January to 26 °C in July. The soil in the area is classified as Gleyic Phaeozems according to the FAO/UNESCO classification system. Throughout the region, artificial afforestation communities, including *Robinia pseudoacacia*(Linn.), *Pinus tabuliformis* (Carr.), and *Platycladus orientalis* (L.) Franco has been established over the past decade. The *R. pseudoacacia* coverage accounts for more than 90%. The dominant understory vegetation are *Stipa bungeana* (Trin.), *Artemisia argyi* (H.) and *Humulus scandens* (Lour.). Coverage of these understory species ranges from 80% to 90% of the vegetation coverage within the study area.

### Experimental design

This study focuses on the first two years of a large precipitation manipulation experiment established in the Qishui watershed on the southern Loess Plateau in spring 2015. Two blocks measuring 125 × 20 m were randomly selected, with one assigned as a throughfall exclusion treatment (drought) and the other as an unaltered control (ambient). Treatments were applied to 20 × 20 m plots. Each plot contains twenty 10 year old black locust trees along the slope. The two treatment blocks were replicated six times for a total of 12 experimental treatment plots. In the drought treatment, polycarbonate sheets were fixed to rails approximately 1 m above the ground from June in 2015 to November in 2016^39,40^ (Fig. S1). This reduced total precipitation by approximately 40% relative to the ambient treatment. Six 30 cm deep ditches were excavated along each treatment plots to converge runoff water from the polycarbonate sheets. Litter falling on the plastic strips was regularly placed below them to ensure that differences in the contents of soil nutrients among treatments were attributable only to the availability of water. In addition, trenches (50 cm deep) were dug around the perimeters of both plots to reduce the lateral inflow of water through the soil matrix from outside the plots.

### Rainfall and soil water content

Rainfall was measured using a tipping bucket rainfall gauge (CS700-L; Campbell Scientific, Logan UT, USA) with a resolution of 0.2 mm in the control plot. Soil volumetric water content (VWC) was measured using electrically conductive sensors (EC-5, Decagon, Pullman WA, USA). The sensors measured changes in the soil dielectric constant within a 240-ml sphere of influence, and are capable of quantifying small changes (0.1%) in VWC. The VWC was estimated from the sensor reading by applying a calibration equation to the scaled frequency output of the capacitance sensors. Within a plot for the drought and ambient treatments, VWC sensors were horizontally installed around the trees at depths of 10, 20, 40, 60, 80, and 100 cm from June in 2015 to November in 2016. The VWC sensors were powered and controlled by a CR1000 data logger (Campbell Scientific, Logan UT, USA) whereby the sensors were scanned every 30 s, and averages were recorded every 10 min.

### Sap flow measurement

The thermal dissipation probe (TDP) method^13^, with a heat source and a TDP sensor, was used to measure sap flow velocity. As a ring-porous species, black locust trees in the study area have relatively narrow sapwood, ranging from 5 to 10 mm^19^. We therefore used 10-mm-long sensors, and the sensors are described in detailed in Peng *et al*.^14^.

Sap flow measurements were conducted from June to September in 2015, and from April to November in 2016. For each treatment, six black locust trees were randomly selected within the same plot, for sap flow measurements using the thermal dissipation method. After peeling off the bark (10 mm × 10 mm), the probes were inserted into the north side sapwood about 10 mm vertically, 1.5 m above the soil surface. The sensors were mounted with waterproof silicone and covered with an aluminum box-cover to avoid solar radiation heating and to prevent exposure to rain. All of the TDP sensors were connected to a CR1000 data logger with an AM16/32 multiplexer (Campbell Scientific, Logan UT, USA). The temperature difference between the upper heated probe and the lower reference probe were scanned every 30 s, and the mean values of 10 min averages were recorded.

Sap flux density on a sapwood area basis was calculated based on the temperature difference between the heated and non-heated probe by an empirical equation^13^:

1 $${Fd}=119\times {10}^{-6}{(\frac{{\Delta }\text{Tmax}-{\Delta }T}{{\Delta }T})}^{1.231}$$

where *F*_*d*_ is sap flux density (m s^−1^); *ΔT* is the temperature difference between the two probes; *ΔT*_*max*_ is the maximum value of Δ*T* when sap flux density is near zero (generally taken between the hours of 02:00 and 05:00 because the sap flux velocity is considered to be minimal during this period^14^.

Several reports suggested that sap flux density is significantly underestimated by the thermal dissipation method^1,17,41^. Therefore, in the present study, we calibrated sap flux densities by the original empirical equation for analyses of their responses to throughfall exclusion^42^.

### Sapwood area and stand transpiration estimation

**Figure 5:**
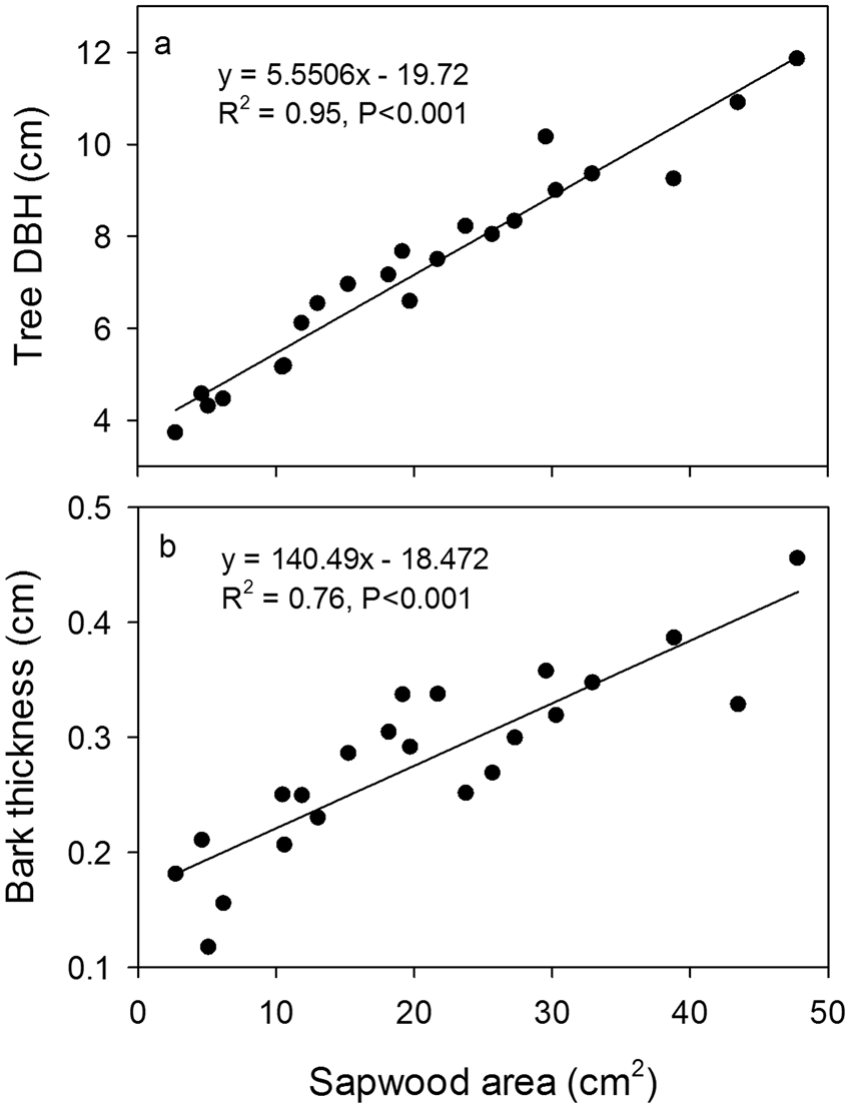
Relationships between tree diameter at breast height (DBH), bark thickness and sapwood area. The solid line is the corresponding regression line fitted using a linear function.

Sapwood area (*A*_*s*_) was a critical factor when calculating the stand transpiration. Before the throughfall exclusion was proceed, core samples were taken from 24 randomly trees near the plots using an increment borer, and discriminating between the sapwood and the heartwood based on color differences. Sapwood area was computed as the difference between the stem cross-sectional area beneath the bark and heartwood area. In this study, regression equations were derived from measurements of cores using tree diameter at breast height (*DBH*) and bark thickness with the sapwood area (Fig. 5). Sapwood area was reliably described as a linear function of *DBH* (*A*_*s*_ = 5.551 * *DBH*−19.72, *R*^2^ = 0.95, *P* < 0.001). Based on the linear regression equation and *DBH* of all trees in these plots, *A*_*s*_ was estimated to range from 8.0 to 67.3 cm^2^ with a mean of 29.1 cm^2^. Total sapwood area (*A*_*st*_) in the throughfall exclusion plot was 0.278 m^2^ (2015), 0.291 m^2^ (2016), and was 0.276 m^2^ (2015) and 0.292 m^2^ (2016) in the ambient plot.

The individual whole-tree sap flow (*F*, m^3^ s^−l^) was calculated as the product of *F*_*d*_ and *A*_*s*_^13^:

2 $$F=Fd\times As$$

Stand transpiration (*Q*, m s^−1^) was extrapolated from the sap flow measurements of individual trees using the equation^22,23^:

3 $$Q=Js\times \frac{Ast}{Ag}$$

Where *A*_*st*_ (m^2^) is the total sapwood area of the plot, *A*_*g*_ is the ground area of the plot (m^2^), and *J*_*s*_ is the average sap flow density of the plot. The variable *J*_*s*_ was calculated as:

4 $$Js={\sum }_{{\rm{i}}=1}^{{\rm{n}}}({\sum }_{{\rm{j}}=1}^{{\rm{k}}}FdijAsij)/Ast$$

where n is the number of *DBH* classes in the plot and k is the number of sample trees in *DBH* class i. The *F*_*dij*_ are the sap flux density of sample tree j in *DBH* class i, and *A*_*sij*_ is the sapwood area for the corresponding sample tree (Table 2). DBH classes was determined at a 2 cm interval according the frequency distribution of tree DBH in each stand.

**Table 2:** Distribution characteristics of sapwood area under drought stress and ambient treatments in 2016. Asi is the total sapwood area in DBH class i, Ast is the ground area of the plot (m^2^).

Diameter (cm)	Drought plot	Ambient plot	A_si_, cm^2^	A_si_/A_st_ %
	A_si_, cm^2^	A_si_/A_st_ %		
6 (5–7)	175.7	14.9	128.0	10.7
8 (7–9)	265.2	22.6	359.3	30.0
10 (9–11)	260.7	22.2	274.8	23.0
12 (11–13)	260.9	22.2	284.8	23.8
14 (13–15)	188.7	16.1	93.4	7.8
15 (>15)	24.0	2.0	56.2	4.7
Total	1175.2	100	1196.7	100

### Stem growth and LAI measurement

An initial inventory of stems ≥5 cm, *DBH* was conducted in April 2015, during which trees were identified to *DBH* was measured. The radial increment was measured using manual band dendrometers (D1, Germany), which were installed at breast height on the 20 selected trees in the two drought and ambient plots, in early June 2015. To ensure a smooth contact between the band and trunk, the outer bark was slightly brushed off. Diameter increment was monitored twice a month from April to November in 2016. The leaf area index (LAI) was estimated indirectly twice a month at these point, where the aluminum neutron-probe access tubes were installed. The LAI was calculated in June - November in 2015 and April - November in 2016 by processing the digital hemispherical photographs using CAN-EYE 4.0^43^.

### Statistical analyses

A T-test was performed to individually test the effects of throughfall exclusion on (1) soil moisture; (2) LAI; (3) monthly stand transpiration, and (4) annual stand transpiration. The relationships between daily and monthly stand transpiration and biological (LAI), edaphic (soil volumetric water content), and environmental characteristics (daily and monthly precipitation) were analyzed. Statistical differences were tested at the *P* < 0.05 level. SigmaPlot 12.5 software for Windows (Systat Software, USA) and SPSS 18.0 software for Windows (SPSS Inc., USA) were used for these analyses.

## Electronic supplementary material

Supplementary Information
