# Supplementary material for: Sap flow of black locust in response to short-term drought in southern Loess Plateau of China
Source: Sci Rep. 2018 Apr 18;8:6222. doi: 10.1038/s41598-018-24669-5 (PMC5906651; doi:10.1038/s41598-018-24669-5)
Supplement: Supplementary file 1 — Supplementary Information [file 41598_2018_24669_MOESM1_ESM.docx]

**Sap flow of black locust in response to short-term drought in southern Loess Plateau of China**

Qingyin Zhang^1^, Xiaoxu Jia^2,3*^, Mingan Shao^1,2,3^, Chencheng Zhang^1^, Xiangdong Li^1^, Changkun Ma^1^

^1^State Key Laboratory of Soil Erosion and Dryland Farming on the Loess Plateau, Northwest A&F University, Yangling 712100, China

^2^Key Laboratory of Ecosystem Network Observation and Modeling, Institute of Geographic Sciences and Natural Resource Research, Chinese Academy of Sciences, Beijing 100101, China

^3^College of Resources and Environment, University of Chinese Academy of Sciences, Beijing, 100190, China

**Corresponding author:**

*XiaoxuJia (jiaxx@igsnrr.ac.cn), Key Laboratory of Ecosystem Network Observation and Modeling, Institute of Geographic Sciences and Natural Resources Research, Chinese Academy of Sciences, Beijing 100101, China.

**
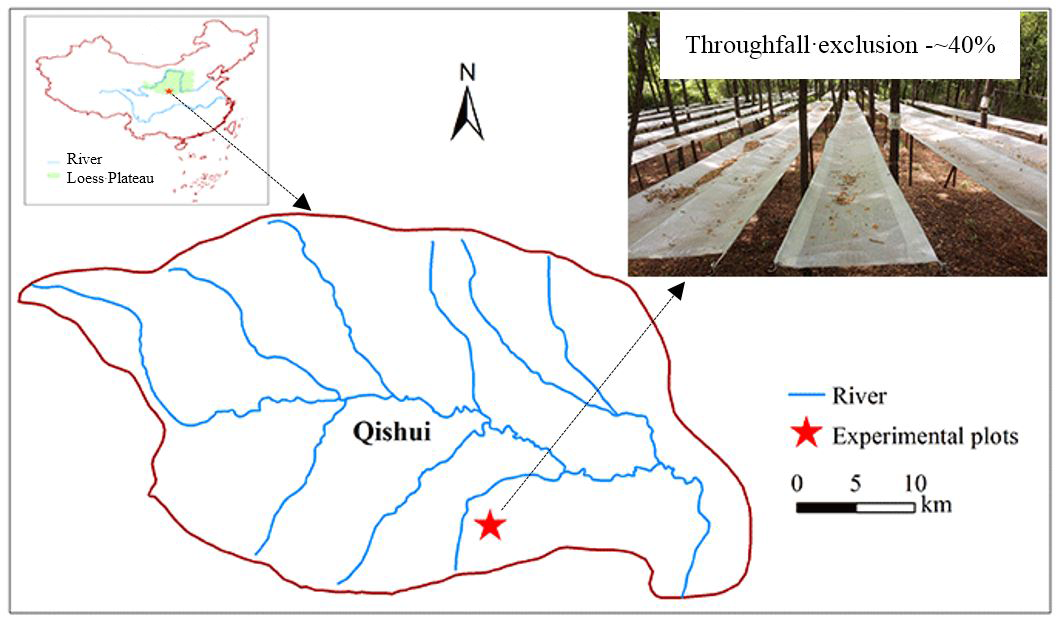
**

**Figure 1S** Location of the study sites on the semi-arid Loess Plateau and a picture of the black locust stand subjected to the throughfall exclusion experiment. (Maps were created using ArcGIS software by ESRI (Environmental Systems Resource Institute, ArcGIS 10.2; *www.esri.com*).
